# Student-Reported School Safety Perceptions, Connectedness, and Absenteeism Following a Multiple-Fatality School Shooting — Broward County, Florida, February 14–21, 2018

**DOI:** 10.15585/mmwr.mm6909a3

**Published:** 2020-03-06

**Authors:** Catherine N. Rasberry, Ganna Sheremenko, Catherine A. Lesesne, India D. Rose, Susan Hocevar Adkins, Lisa C. Barrios, Kristin M. Holland, Valerie Sims, Kevin O’Connor, Dominic J. Grasso, Sebrina R. James, Thomas R. Simon

**Affiliations:** ^1^Division of Adolescent and School Health, National Center for HIV/AIDS, Viral Hepatitis, STD, and TB Prevention, CDC; ^2^ICF International, Atlanta, Georgia; ^3^Division of Violence Prevention; National Center for Injury Prevention and Control, CDC; ^4^Department of Equity and Diversity, Broward County Public Schools, Fort Lauderdale, Florida.

From July 2009 to June 2018, the rates of multiple-victim, school-associated homicides in the United States fluctuated substantially, with evidence of a significant increase in recent years (*1*). Data on the effects of such incidents on students’ school attendance and perceptions of safety and connectedness are limited (*2*,*3*) but important. This study used data from a neighboring within-district school before and after a multiple-fatality shooting at Marjory Stoneman Douglas High School in Parkland, Florida, on February 14, 2018. Self-administered questionnaires were completed by one group of students on February 14 just before the shooting (575) and another group during February 15–21 (502); demographics for these groups appeared similar. Linear and logistic regression analyses controlling for demographic characteristics explored differences between groups for safety-related perceptions or experiences, school connectedness, and absenteeism. Compared with students surveyed before the shooting, students surveyed in the days immediately following the shooting had lower odds of feeling safe at school, higher odds of absenteeism, and higher school connectedness scores. Findings suggest the shooting had an immediate, sizeable effect on safety perceptions and absenteeism among students in a neighboring school. Findings also suggest higher school connectedness following the shooting. Further study of school connectedness, including how to enhance and sustain it, might help schools and communities better respond to traumatic events in the community.

Data were collected from a census of students in one high school participating in an ongoing evaluation project in Broward County Public Schools. Data collection was to be split over 2 days, February 14–15, 2018; however, Marjory Stoneman Douglas High School, a within-district neighboring school, experienced a school shooting resulting in 17 homicides and 17 additional persons injured on February 14.* Data collection that day was completed before the shooting occurred. Remaining data collection, originally scheduled for February 15, occurred February 15–21 at the discretion of school administration.

Teachers proctored a 47-item, voluntary, anonymous, paper-and-pencil questionnaire during personalization periods.^†^ Approximately half the periods received questionnaires as scheduled on February 14, and remaining periods completed questionnaires within 1 week. Passive parental consent forms were sent home in advance; students who did not assent or whose parents opted them out did not participate. Response rates, calculated from enrollment, were 49.0% overall (53.1% and 44.9% for February 14 and 15–21, respectively). Questionnaires missing >25% of responses (29) were not analyzed. The Institutional Review Board at ICF, the research and evaluation firm contracted to conduct the original evaluation, approved the project, following CDC ethics guidelines.^§^

This analysis focuses only on responses to questions about safety-related perceptions/experiences, school connectedness, and absenteeism from a larger questionnaire. Safety-related indicators included feeling safe at school and avoiding school spaces because of feeling uncomfortable or unsafe. School connectedness was measured by the average score of a 5-item scale (range = 1–5; 5 reflects greatest connectedness), based on a valid and reliable school connectedness scale used elsewhere (*4*). Scale indicators included feeling close to people at school, feeling accepted and belonging at school, feeling happy at school, believing staff members at school treat students fairly, and believing staff members at school care about them. Responses were dichotomized, reflecting responses of “strongly agree” or “agree” for individual indicator analysis. Absenteeism was assessed with two indicators: that the student did not go to school for ≥1 day in the past 30 days, and that the student did not go to school for ≥1 day in the past 30 days because of feeling unsafe.

Variable frequencies were calculated for students surveyed before the shooting and those surveyed after. Chi-squared tests and a t-test assessed differences between administration groups. Logistic and linear regression models adjusting for sex, age, and race/ethnicity tested differences between groups for all safety, connectedness, and absenteeism variables.^¶^ Analyses were conducted using SPSS (Statistics Subscription; IBM).

Participants comprised 1,077 students, including 575 (53.4%) surveyed before the shooting and 502 (46.6%) surveyed after. Chi-squared tests revealed no significant demographic differences between students surveyed before and after the shooting, with a slight overrepresentation of Hispanic students (Table 1); however, there were significant differences for one of two safety-related variables, three of five school connectedness variables, and both absenteeism variables. In addition, a t-test revealed a significant difference in average school connectedness. Differences were further explored through adjusted regression models (Table 2). Logistic regressions revealed that students surveyed after the shooting, compared with those surveyed before, had significantly lower odds of feeling safe at school (adjusted odds ratio [AOR] = 0.48; 95% confidence interval [CI] = 0.36–0.63), but significantly higher odds of reporting feeling happy at school (AOR = 1.58; 95% CI = 1.23–2.02), believing staff members at school treat students fairly (AOR = 1.46; 95% CI = 1.14–1.87), and believing staff members at school care about them (AOR = 1.38; 95% CI = 1.08–1.76). In addition, students surveyed after the shooting had significantly higher odds of not going to school for ≥1 day in the past 30 days (AOR = 2.06; 95% CI = 1.55–2.74) and missing school ≥1 day in the past 30 days because they felt unsafe (AOR = 7.18; 95% CI = 4.87–10.60). Linear regression results found that students surveyed after the shooting had significantly higher average school connectedness scores (mean = 3.35) than those before the shooting (mean = 3.22) (Table 1) (regression coefficient [B] = 0.125; standard error = 0.05; 95% CI = 0.03–0.22) (Table 2).

**TABLE 1:** Demographic characteristics and safety-related perceptions/experiences, school connectedness, and absenteeism characteristics of students surveyed before and after a school shooting — 2018 Youth Health and School Climate Survey, Broward County, Florida, February 14–21, 2018

Characteristic	No. (%)		Chi-squared or t-test results^§^	p-value
	Students surveyed before the shooting (n = 575)*	Students surveyed after the shooting (n = 502)^†^		
**Sex**				
Female	288 (50.3)	253 (50.6)	0.01	0.91
Male	285 (49.7)	247 (49.4)		
**Age (yrs)**				
≤12	6 (1.0)	2 (0.4)	6.63	0.36
13	0 (0.0)	2 (0.4)		
14	70 (12.2)	74 (14.7)		
15	151 (26.4)	133 (26.5)		
16	137 (24.0)	123 (24.5)		
17	141 (24.7)	106 (21.1)		
≥18	67 (11.7)	62 (12.4)		
**Race/Ethnicity**				
Black, non-Hispanic	153 (26.9)	125 (25.1)	5.13	0.16
Hispanic	337 (59.3)	287 (57.5)		
White, non-Hispanic	45 (7.9)	40 (8.0)		
Other or multiracial, non-Hispanic	33 (5.8)	47 (9.4)		
**Safety-related perceptions/experiences**				
Feel safe at school^¶^	437 (78.7)	313 (64.4)	26.44	<0.001
Avoid spaces at school attributable to feeling uncomfortable or unsafe**	85 (16.2)	73 (15.8)	0.02	0.88
**School connectedness**				
Feel close to people at school^††^	264 (46.2)	225 (45.3)	0.08	0.77
Feel accepted and like I belong at school^§§^	300 (52.7)	283 (56.6)	1.61	0.20
Feel happy at school^¶¶^	218 (38.4)	249 (49.7)	13.69	<0.001
Staff members at school treat students fairly***	222 (39.4)	240 (48.4)	8.74	<0.01
Staff members at school care about me^†††^	246 (43.7)	254 (51.4)	6.30	0.01
Average school connectedness score, mean (SD)^§§§^	3.22 (0.78)	3.35 (0.75)	2.65	<0.01
**Absenteeism**				
Did not go to school for ≥1 day in the past 30 days, mean (SD)^¶¶¶^	373 (65.7)	391 (79.1)	23.79	<0.001
Did not go to school for ≥1 day in the past 30 days because of feeling unsafe, mean (SD)****	41 (11.0)	178 (46.2)	114.09	<0.001

**Abbreviation:** SD = standard deviation.

* Students were surveyed on February 14, the day of, but before, the shooting at another school in the district.

^†^ Students were surveyed after February 14 and within 1 week of the shooting at another school in the district.

^§^ School connectedness differences tested with a t-test; all other differences tested using chi-squared tests. Statistical tests were considered significant if p<0.05.

^¶^ Question asked: “Do you feel safe at your school?” (response options: yes and no).

** Question asked: “Do you avoid spaces at school because you feel uncomfortable or unsafe in the space?” (response options: yes and no).

^††^ Reflects responses of “strongly agree” or “agree” to the statement “I feel close to people at this school” (response options: strongly agree, agree, neither agree nor disagree, disagree, or strongly disagree).

^§§^ Reflects responses of “strongly agree” or “agree” to the statement “I am accepted and feel like I belong at this school” (response options: strongly agree, agree, neither agree nor disagree, disagree, or strongly disagree).

^¶¶^ Reflects responses of “strongly agree” or “agree” to the statement “I feel happy at this school” (response options: strongly agree, agree, neither agree nor disagree, disagree, or strongly disagree).

*** Reflects responses of “strongly agree” or “agree” to the statement “Staff (such as a teacher, counselor, nurse, coach, or other school staff) at this school treats students fairly” (response options: strongly agree, agree, neither agree nor disagree, disagree, or strongly disagree).

^†††^ Reflects responses of “strongly agree” or “agree” to the statement “Staff (such as a teacher counselor, nurse, coach, or other school staff) at this school care about me” (response options: strongly agree, agree, neither agree nor disagree, disagree, or strongly disagree).

^§§§^ Overall school connectedness score is an average of the 5 school connectedness items (range = 1–5). Scores >3 reflect a positive perception of school connectedness.

^¶¶¶^ Question asked: “During the past 30 days, on how many days did you not go to school” (response options: 0 days, 1 day, 2 or 3 days, and 4 or more days).

**** Question asked: “During the past 30 days, on how many days did you not go to school because you felt unsafe at school or on your way to or from school?” (response options: 0 days, 1 day, 2 or 3 days, and 4 or more days).

**TABLE 2:** Association between survey administration time point* and safety-related perceptions/experiences, school connectedness, and absenteeism, adjusted for sex, age, and race/ethnicity (logistic and linear regression analyses^†^) — 2018 Youth Health and School Climate Survey, Broward County, Florida, February 14–21, 2018

Characteristic	AOR or B (SE)	(95% CI)	p-value
**Logistic regression results**^†^ **(AOR)**			
**Safety-related perceptions/experiences**			
Feel safe at school^§^	0.48	(0.36–0.63)	<0.001
Avoid spaces at school attributable to feeling uncomfortable or unsafe^¶^	0.95	(0.67–1.34)	0.76
**School connectedness**			
Feel close to people at school**	0.97	(0.76–1.24)	0.80
Feel accepted and like I belong at school^††^	1.18	(0.92–1.51)	0.19
Feel happy at school^§§^	1.58	(1.23–2.02)	<0.001
Staff members at school treat students fairly^¶¶^	1.46	(1.14–1.87)	<0.01
Staff members at school care about me***	1.38	(1.08–1.76)	0.01
**Absenteeism**			
Did not go to school 1 or more days in the past 30 days^†††^	2.06	(1.55–2.74)	<0.001
Did not go to school 1 or more days in the past 30 days because of feeling unsafe^§§§^	7.18	(4.87–10.60)	<0.001
**Linear regression results**^†^ **(B [SE])**			
**School connectedness**			
Average school connectedness score	0.13 (0.05)	(0.03–0.22)	<.01

**Abbreviations:** AOR = adjusted odds ratio; B = regression coefficient; CI = confidence interval; SE = standard error.

* For the first administration, students were surveyed on February 14 (the day of, but before, the shooting at another school in the district). For the second administration, students were surveyed after February 14 and within 1 week of the shooting at another school in the district.

^†^ Regressions controlled for sex, age (used as a continuous variable), and race/ethnicity (four categories, with white, non-Hispanic as the referent). Statistical tests were considered significant if p<0.05. The administration time point indicator was coded as zero for students surveyed before the shooting (on February 14; referent group), and 1 for students surveyed after the shooting (after February 14).

^§^ Question asked: “Do you feel safe at your school?” (response options: yes and no).

^¶^ Question asked: “Do you avoid spaces at school because you feel uncomfortable or unsafe in the space?” (response options: yes and no).

** Reflects responses of “strongly agree” or “agree” to the statement “I feel close to people at this school” (response options: strongly agree, agree, neither agree nor disagree, disagree, or strongly disagree).

^††^ Reflects responses of “strongly agree” or “agree” to the statement “I am accepted and feel like I belong at this school” (response options: strongly agree, agree, neither agree nor disagree, disagree, or strongly disagree).

^§§^ Reflects responses of “strongly agree” or “agree” to the statement “I feel happy at this school” (response options: strongly agree, agree, neither agree nor disagree, disagree, or strongly disagree).

^¶¶^ Reflects responses of “strongly agree” or “agree” to the statement “Staff (such as a teacher, counselor, nurse, coach, or other school staff) at this school treats students fairly” (response options: strongly agree, agree, neither agree nor disagree, disagree, or strongly disagree).

*** Reflects responses of “strongly agree” or “agree” to the statement “Staff (such as a teacher counselor, nurse, coach, or other school staff) at this school care about me” (response options: strongly agree, agree, neither agree nor disagree, disagree, or strongly disagree).

^†††^ Question asked; “During the past 30 days, on how many days did you not go to school” (response options: 0 days, 1 day, 2 or 3 days, and 4 or more days).

^§§§^ Question asked: “During the past 30 days, on how many days did you not go to school because you felt unsafe at school or on your way to or from school?” (response options: 0 days, 1 day, 2 or 3 days, and 4 or more days).

## Discussion

From July 2009 to June 2018, rates of multiple-victim, school-associated homicides increased in the United States (*1*), yet data surrounding these events are limited. Findings of this study provide unique insight into students’ perceptions and experiences following a school shooting. Findings revealed an immediate, detrimental difference to perceived school safety and attendance among students following a shooting in a nearby school. Compared with students surveyed before the shooting, students surveyed after the shooting had approximately one half the odds of reporting feeling safe at school, twice the odds of reporting general absenteeism, and seven times the odds of reporting absenteeism because they felt unsafe.

Other research has shown that students’ fear and absenteeism were higher after the 1999 Columbine school shooting (*2*,*3*). These studies, using national samples, reported generally consistent findings, but of a smaller magnitude than the current study’s findings. The larger magnitudes in this study might be partially explained by closer temporal and physical proximity of students to the event, because physical proximity to or social distance from traumatic events influences their impact (*2*,*5*).

These findings show that a school shooting’s effects extend beyond the school where it occurred. Students could have been influenced by factors such as degree of exposure, media coverage, number of victims known, and perceived similarity to victims, which have been associated with general distress and acute stress immediately following traumatic events (*5*).

Results also suggest possible strengthening of overall school connectedness and three of five connectedness indicators. Students surveyed after the shooting had 37%–57% higher odds of reporting feeling happy at school, that school staff members cared about them, and that school staff members treated students fairly. This aligns with literature documenting increased social solidarity following traumatic events that impact communities collectively (*6*,*7*). Following the shooting, the studied school gave students opportunities to discuss the incident with classmates and staff members. The school implemented an open-door policy for students and staff members to visit administrators or counselors at any time, fostered efforts of student-led clubs and organizations to support Marjory Stoneman Douglas High School students and staff members, and explored strategies to make their own school safer. These opportunities might have fostered increased connectedness, which might provide, at least in the short term, a protective buffer against negative posttrauma impacts. Activating existing support networks can help support individuals following trauma (*8*), and promoting connectedness can have numerous benefits,** including a beneficial effect on youths’ risk for interpersonal violence and suicide (*9*).

The findings in this study are subject to at least four limitations. First, cross-sectional data do not allow before and after comparisons of the same students or long-term follow-up. Second, students could not be randomly assigned to “before” or “after” conditions; however, demographic characteristics of the two administration groups were similar. Third, data collection might have been affected by students’ absences attributable to the shooting. Questionnaire administration records estimate absenteeism of 28% and 33% during the first and second administration groups, respectively. Connectedness estimates could be inflated if less connected students were absent, and absenteeism and safety-related findings might be underestimates. Finally, the overall response rate of <50% could affect generalizability of the findings. Compared with enrollment records, the sample’s demographic patterns were similar to that of the school.

Despite these limitations, this study has important strengths. It reports on school connectedness, a construct yet to be examined following school shootings. Furthermore, the studied sample comprises primarily black and Hispanic students, rather than predominately white students as often has been found in studies following similar events (*8*).

Collectively, findings underscore the immediate, detrimental effect on students’ safety perceptions and absenteeism following a multiple-fatality shooting at a neighboring school, suggesting trauma-informed supports might be beneficial for students attending schools near sites of school shootings. Findings also suggest a measurable shift in school connectedness following the shooting, possibly from formal and informal efforts to provide, and spontaneous instances of, social support and solidarity, which might buffer trauma-related impacts. Further study of school connectedness, including how to enhance and sustain it, might help schools and communities better respond to traumatic events in the community.

**Summary**

**What is already known about this topic?**

Limited research has shown increases in students’ fear and absenteeism in the aftermath of school shootings. However, no study has examined students in an affected district immediately before and after a school shooting.

**What is added by this report?**

Detrimental changes to perceived school safety and absenteeism and an increase in school connectedness were identified among Florida high school students in one school immediately following a shooting at a nearby school.

**What are the implications for public health practice?**

Findings suggest a need for trauma-informed supports for students attending schools near sites of school shootings. Increasing school connectedness, through formal and informal efforts, in addition to spontaneous instances of social support and solidarity, might help buffer trauma-related impacts.

## Footnotes

*
https://www.npr.org/2019/02/14/694688365/we-live-with-it-every-day-parkland-community-marks-one-year-since-massacre .

† Personalization periods are similar to traditional study halls and are designated class periods in which all students are expected to enroll.

§ https://www.cdc.gov/os/integrity/hrpo/regAndGuidance.htm.

¶ Average school connectedness was examined using a t-test and linear regression. All other variables, including specific school connectedness indicators, were examined using chi-squared tests and logistic regression models.

**
https://www.cdc.gov/healthyyouth/protective/school_connectedness.htm .
